# Exosome-based immunotherapy: a promising approach for cancer treatment

**DOI:** 10.1186/s12943-020-01278-3

**Published:** 2020-11-12

**Authors:** Zhijie Xu, Shuangshuang Zeng, Zhicheng Gong, Yuanliang Yan

**Affiliations:** 1 Department of Pathology, Xiangya Hospital, Central South University, Changsha, 410008 Hunan China; 2 Department of Pharmacy, Xiangya Hospital, Central South University, 87 Xiangya Road, Changsha, 410008 Hunan China; 3 National Clinical Research Center for Geriatric Disorders, Xiangya Hospital, Central South University, Changsha, 410008 Hunan China

**Keywords:** Exosomes, Cancer immunotherapy, Cancer vaccines, Immune cells, Clinical implications

## Abstract

In the era of the rapid development of cancer immunotherapy, there is a high level of interest in the application of cell-released small vesicles that stimulate the immune system. As cell-derived nanovesicles, exosomes show great promise in cancer immunotherapy because of their immunogenicity and molecular transfer function. The cargoes carried on exosomes have been recently identified with improved technological advances and play functional roles in the regulation of immune responses. In particular, exosomes derived from tumor cells and immune cells exhibit unique composition profiles that are directly involved in anticancer immunotherapy. More importantly, exosomes can deliver their cargoes to targeted cells and thus influence the phenotype and immune-regulation functions of targeted cells. Accumulating evidence over the last decade has further revealed that exosomes can participate in multiple cellular processes contributing to cancer development and therapeutic effects, showing the dual characteristics of promoting and suppressing cancer. The potential of exosomes in the field of cancer immunotherapy is huge, and exosomes may become the most effective cancer vaccines, as well as targeted antigen/drug carriers. Understanding how exosomes can be utilized in immune therapy is important for controlling cancer progression; additionally, exosomes have implications for diagnostics and the development of novel therapeutic strategies. This review discusses the role of exosomes in immunotherapy as carriers to stimulate an anti-cancer immune response and as predictive markers for immune activation; furthermore, it summarizes the mechanism and clinical application prospects of exosome-based immunotherapy in human cancer.

## Background

Cancer is a major public health problem and the leading cause of death globally, and cancer incidence and mortality are rapidly growing worldwide. More than 18 million new cancer cases and 9 million cancer deaths are currently expected each year [1–3]. Common cancer treatments mainly include surgery, chemotherapy, radiotherapy and targeted therapy [4]. However, chemotherapy and/or radiotherapy, as the most important and effective therapeutic strategies for treating cancer, can also cause adverse reactions, drug resistance and long-term complications [5, 6]. Given the significant advances in drug screening technology, there is now emerging interest in oncology drug development that can overcome these problems by using a new cancer therapy strategy [7, 8]. Cancer immunotherapy is type of a treatment that controls and clears tumors by regulating the immune system to reactivate the anti-cancer immune response and overcome the pathway leading to tumor escape [9, 10]. Therapeutic approaches mainly include nonspecific immune stimulation, immune checkpoint blockades, adoptive cell transfer and vaccination strategies. Several immunotherapy drugs have been approved by the United States Food and Drug Administration (FDA) for clinical use, such as cytotoxic T-lymphocyte-associated protein 4 (CTLA-4) inhibitors, programmed cell death 1 (PD-1) inhibitors and programmed cell death 1 ligand 1 (PD-L1) inhibitors [11–13]

Exosomes are single-membrane organelles with a diameter of approximately 30–100 nm that can be secreted by many types of cells, including cancer cells and immune cells [14]. The main molecular components of exosomes are cell-derived proteins, lipids, glycoconjugates and nucleic acids [15, 16]. Exosomes have a variety of activities such as remodeling the extracellular matrix (ECM) as well as mediating the intercellular transmission of signals and molecules. With the study of multiple roles of exosomes in cancer progression, the dual characteristics of exosomes in promoting and suppressing cancer have been considered. As cell-derived nanovesicles, exosomes have potential uses in cancer immunotherapy because of their immunogenicity and molecular transfer functions [17].

In recent years, cancer immunotherapy has become a research hotspot because of its characteristics of strengthening the immune system, applicability to a variety of cancers, and an enduring response. It has shown strong anti-tumor activity in a variety of tumors, including melanoma, non-small cell lung cancer (NSCLC), and kidney cancer [18–20]. Exosomes released by cancer cells can alter different types of stromal cells to promote cancer cell growth and invasive behavior and to activate autocrine VEGF signaling in endothelial cells to promote tumor angiogenesis [21, 22]. Moreover, exosomes can also express molecules that mediate immunosuppression, such as PD-L1 and transforming growth factor-β (TGF-β) [23]. Cancer-derived exosomes can inhibit the proliferation and activation of CD8+ T cells and promote the expansion of regulatory T cells to play an immunosuppressive role [24]. Moreover, some surprising anti-cancer functions of exosomes have recently been revealed. Many studies have found that dendritic cell (DC)- and tumor-derived exosomes express a large number of major histocompatibility complex class I molecules (MHC I) and tumor markers such as heat shock proteins (HSP), which are involved in antigen presentation and stimulation of T cells and have been shown to trigger CD8+ T cell-dependent anti-tumor responses *in vitro* and *in vivo* [25]. Therefore, as carriers to stimulate anti-cancer immune responses and deliver anti-cancer drugs, how exosomes could be utilized in immune therapy is important in regards to cancer progression and they have implications for diagnostics and the development of novel therapeutic strategies. In this review, we focused on the function and mechanism of exosome-based immunotherapy in human cancer, its significant therapeutic effect on cancer progression and the possibility of developing immunotherapeutic vaccines.

## The regulatory role of exosome-based immune responses

**Fig. 1:**
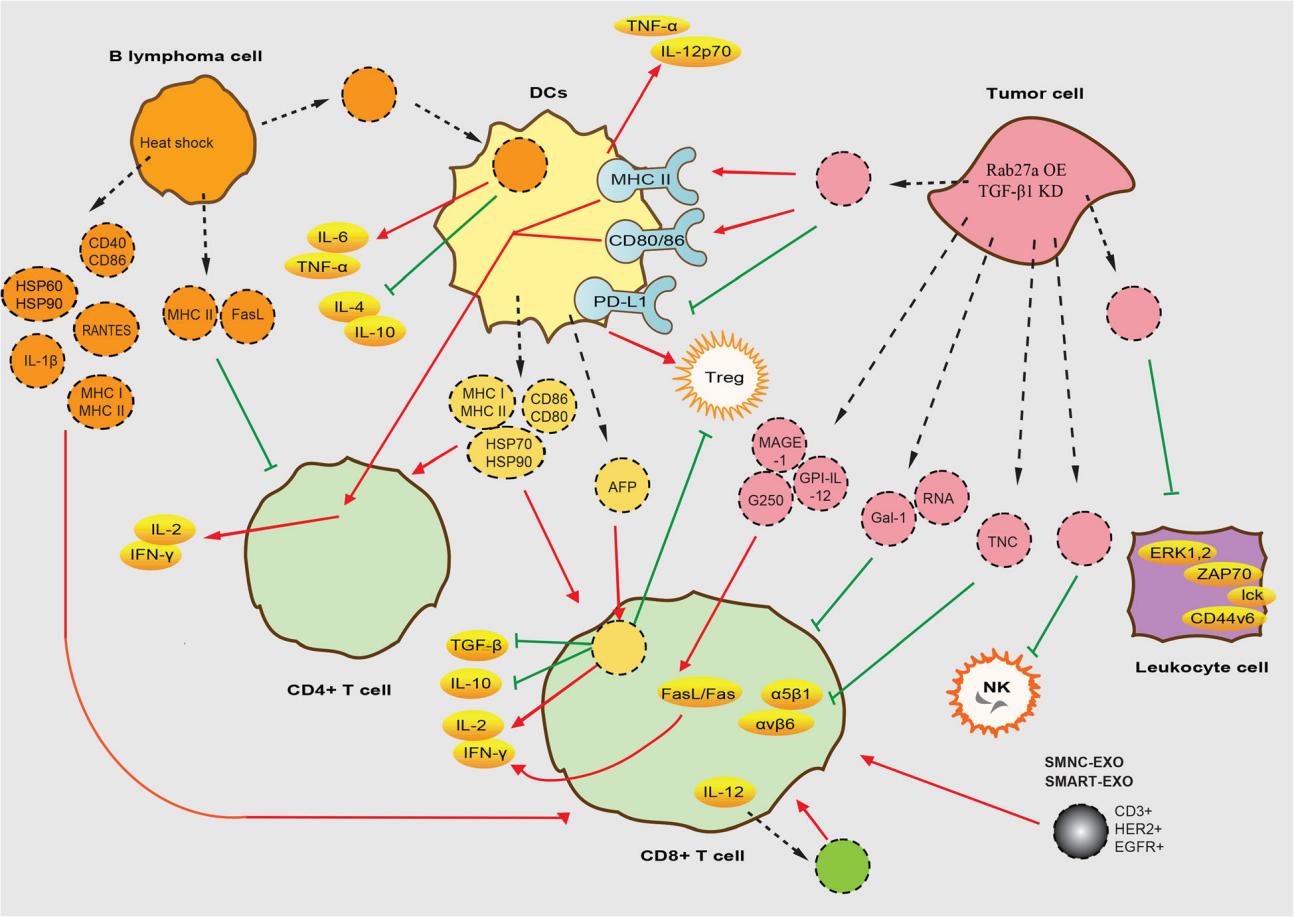
Regulatory mechanisms of exosomes released by different cells on immune cells. Exosomes’ entry and exit into cells is indicated by black dotted lines. Exosomes are represented with the same color as the host cell. OE: overexpression. KD: knock-down

**Fig. 2:**
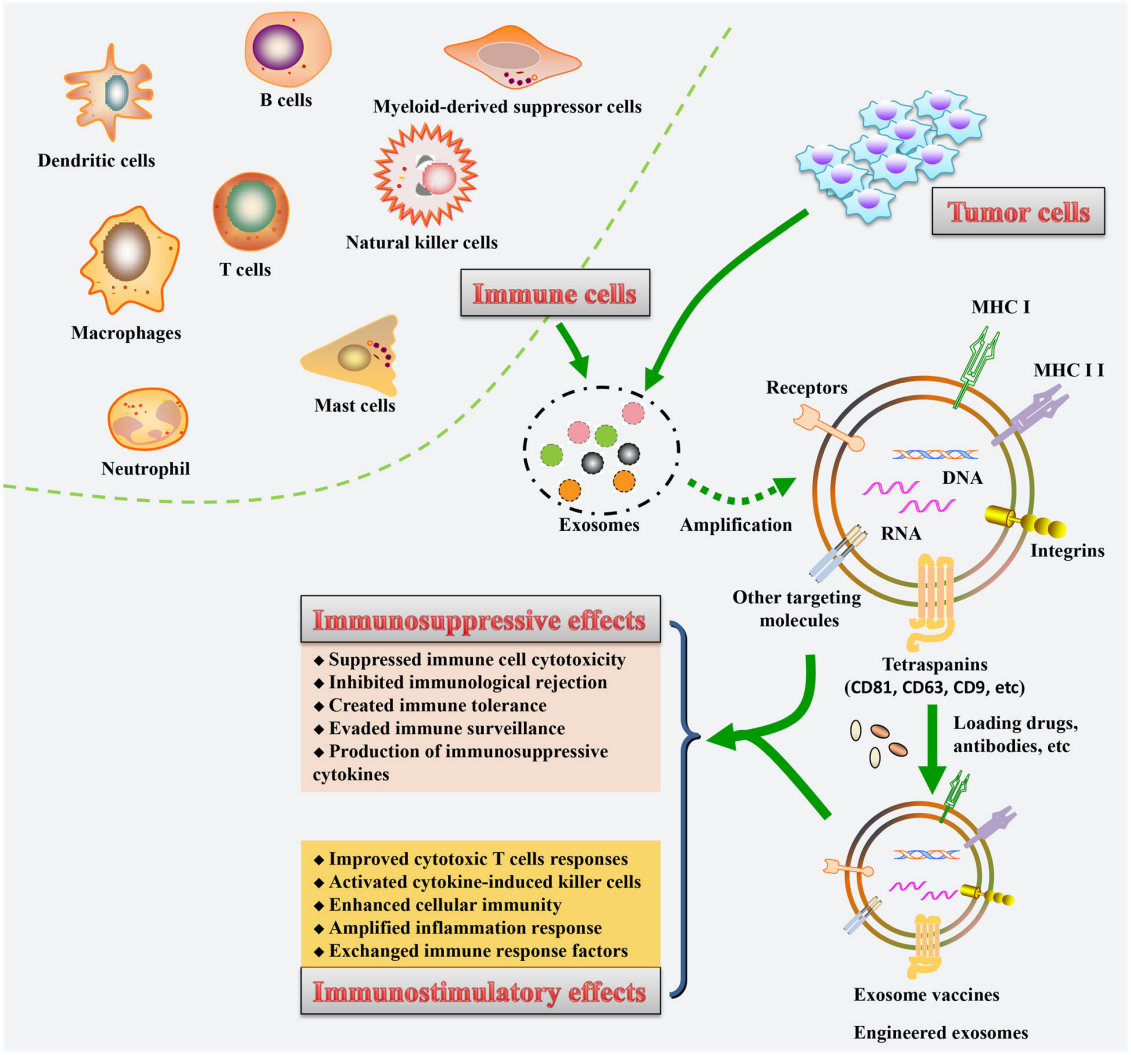
The immune stimulatory and suppressive effects of cells-derived exosomes. This schematic displays the underlying mechanisms and functions of exosomes released from tumor cells and immune cells in the regulation of immune responses in tumor-bearing hosts

The immune response refers to the body's defensive response to harmful substances that are foreign or self-mutated. The immune response can be divided into the innate immune response and the adaptive immune response. Different types of immune cells are involved in the above nonspecific and specific immune responses. Phagocytes (including monocytes, macrophages and DCs) and natural killer (NK) cells are involved in innate immunity and constitute the first line of defense against pathogens; they also synergistically participate in the adaptive immune response. The adaptive acquired immune response utilizes T and B lymphocytes and their immunoglobulins and cytokines to produce a specific and heterogeneous response to invading microorganisms [26–28]. Currently, efforts are being made in the field of immunotherapy to find new low-toxicity inhibitors and better biosafety delivery vectors. Therefore, exosome-based therapy is a potential new approach to cancer immunotherapy because exosomes can be used as carriers to initiate anti-cancer immune responses and as a tool to deliver anti-cancer drugs [29] (Fig. 1). In the following chapter, the immune stimulatory and suppressive effects of exosomes secreted from different cells will be explained in detail (Fig. 2).

### Tumor-released exosomes

Tumor-released exosomes have been widely studied in various types of cancer, such as renal cancer, hematological cancer, breast cancer and melanoma. Tumor-associated exosomes (TAEs) have essential roles in DCs participating in anti-cancer immune responses. Cooperating with DCs, exosomes from a rat pancreatic adenocarcinoma can activate tumor-antigen-specific cytotoxic T cell (CTL) responses and affect leukocyte proliferation through reduced CD44v6 upregulation and lck, ZAP70 and ERK1,2 phosphorylation [30]. A study of pancreatic cancer later found that miRNA-depleted exosome proteins may act as agonists for specifically activating DC/cytokine-induced killer cells (DC/CIK) [31]. In research on NSCLC, exosomes from Rab27a-overexpressing tumor cells have been shown to promote the maturation of DCs by upregulating major histocompatibility complex class I molecules (MHC II) and the costimulatory molecules CD80 and CD86, significantly promoting the proliferation and response of CD4+ T cells *in vitro* and *in vivo* [32]. More importantly, TAEs decreased the expression of PD-L1 on DCs, leading to the downregulation of Tregs *in vitro* [33]. In addition to upregulating MHC II and costimulatory molecules, TGF-β1-silenced leukemia cell-derived exosomes promote DC function by inducing the secretion of interleukin (IL)-12p70 and tumor necrosis factor (TNF)-α [34].

The purpose of cancer immunotherapy is to promote the activity of intracellular CTLs, assist in the initiation of tumor-specific CTLs in lymphoid organs, and establish effective and lasting anti-cancer immunity; thus, CD8+ T cells are the key to controlling cancer [35]. In immunotherapy, by ensuring the transmission of signals from CD4+ T cells to CD8+ T cells and regulating the metabolic activities of T cells, the CTL response can be optimized, which may enhance anti-cancer immunity [36]. In renal cancer, exosomes derived from glycolipid-anchored-IL-12 (GPI-IL-12) gene-modified tumor cells express the tumor-associated antigen MAGE-1 and tumor rejection antigens G250 and GPI-IL-12, which significantly promote T cell proliferation and increase interferon (IFN)-γ in turn, and efficiently trigger a stronger activity of CTLs through the FasL/Fas signaling pathway [37, 38]. Breast cancer exosomes inhibit both CD8+ and CD4+ T cell proliferation by initiating cell apoptosis and suppressing NK cell cytotoxicity and, hence, may inhibit the anticancer immune response [39]. In head and neck cancer, TAEs have been shown to induce a suppressor phenotype in CD8+ T cells in the synergistic action of exosomal proteins such as galectin-1 (Gal-1) and RNA [40]. Exosomes derived from B16F0 melanoma cells suppress cytotoxic immunity by altering the transcriptome of CTLs so that their mitochondrial respiration is not dependent on substrates or hypoxia [41]. Subsequent studies confirmed that in leukemia cell derived exosomes, silencing exosomal TGF-β1, which reduces the level of immunogenicity, can promote CD4+ T cell proliferation and Th1 cytokine (IFN-γ and IL-2) secretion, effectively stimulating the CTL response and the cytotoxicity of NK cells [34]. Brain tumor-initiating cells secrete exosomes for the output of ECM protein tenascin-C, which can inhibit the proliferation of T cells by interactions of α5β1 and αvβ6 integrins associated with the reduction of mTOR signal transduction [42]. In addition, exosomes secreted by mesenchymal stem cells have been investigated to promote the proliferation and immunosuppressive capacity of Tregs by upregulating IL-10 and TGF-β1 in peripheral blood mononuclear cells (PBMCs), and they may play an immunomodulatory role in PBMCs from asthmatic patients through the antigen presenting cell (APC)-dependent pathway [43].

There is ample evidence that TAEs bearing NK ligands are usually able to evade immune surveillance and responses [44, 45]. As reported in the literature, NK cells in host immunity against cancers are predominantly mediated by active receptors, such as NKG2D, NKp44, etc [44]. However, TAEs from tumor cell supernatants and sera of leukemia patients decrease the cytotoxic activity of host NK cells by shedding NKG2D, thereby subverting the host immune system and contributing to the tumor-promoting microenvironment [46, 47]. Similarly, exosomes produced by human solid cancers, including prostate cancer [48] and ovarian cancer [49], can selectively downregulate NKG2D levels on NK cells by expressing NKG2D ligands, ultimately leading to impaired NK cell-mediated cytotoxic function and promotion of tumor immune evasion. In addition, it was demonstrated that TGF-β1, serving as a major immunosuppressive cytokine, restrains the cytolytic effect of NK cells through activation of the Smad2/3 signaling pathway [50]. A subsequent study by Zhao et al. showed that TAEs can induce Smad2/3 phosphorylation in NK cells and attenuate NK cell cytotoxicity against pancreatic cancer stem cells [51]. Unexpectedly, some contrary findings revealed that exosomes originating from HSO70/BAG4-positive tumor sublines have been shown to stimulate the killing effect of NK cells against HSP70-positive tumors [52]. Additionally, in response to genotoxic stress signals, some malignancies release BAG6+ or HSP70+ exosomes and promote NK cell-mediated anti-tumor responses by engaging the active receptors CD69, NKG2D, NKp44 and NKp46 [53–55]. Thus, depending on their cellular origin and environmental conditions, TAEs might display different functional roles in the NK cell-dependent immune response to tumors, which needs more clarification in the future.

### Dendritic cell-derived exosomes

DCs play an important role in tumor immunity due to their ability to absorb and express tumor-associated antigens, and they are important targets in cancer immunotherapy. However, their anti-tumor effect has been unsatisfactory due to the poor immunogenicity of tumor antigens, low uptake efficiency of antigens, and the activation of regulatory T cells [56]. At present, studies have reported that exosomes can be used as the ideal antigen for DC vaccines [57]; thus, it is necessary to explore the mechanism of anti-tumor immunity induced by exosome-based DC vaccines and then confirm whether exosomes can be used as tumor antigens for DC vaccine-based immunotherapy.

As the most effective antigen-presenting cell, DCs also secrete a large number of exosomes to induce effective anticancer effects. DC-derived exosomes (DEX), containing MHC I, MHC II, CD86 and HSP70-90 chaperones, are able to trigger CD4+ and CD8+ T cell activation [58, 59]. Under the costimulation of secreted IL-2 and exosomal CD80, the expression of exosomal peptide MHC I is passed to CD8+ T cells, thereby stimulating the proliferation of CD8+ T cells and inducing more effective anti-tumor immunity *in vivo* [60]. Additional studies have verified that DEX activates CD8+ and CD4+ T cells and induces an anti-tumor immune response by exosomal CD80 and endogenous IL-2 *in vivo* [61, 62]. In addition, exosomes derived from α-fetoprotein (AFP)-expressing DCs stimulated mice with hepatocellular carcinoma to produce more IFN-γ-expressing CD8+ T cells, with increased IFN-γ and IL-2 and reduced CD25+Foxp3+Tregs, IL-10 and TGF-β [63]. Although it is widely believed that DEX containing MHC promotes T cell responses [64], it is controversial that some studies have found that the T cell response can be independent of the MHC contained in DEX if whole antigens are present [65].

### B lymphoma cell-derived exosomes

It has been reported that exosome-based DC vaccines can stimulate clonal expansion of T cells by pulses of exosomes derived from diffuse large B cell lymphoma cells [66]. In contrast, exosomes from B cell lymphoma cells have been found to induce apoptosis in CD4+ T cells via MHC II and FasL [67]. Exosomes secreted by B lymphoma cells subjected to heat shock contained more HSP60 and HSP90 and exhibited an increased levels of immunogenicity molecules, such as MHC I, MHC II, CD40, CD86, RANTES and IL-1β, thus effectively activating CD8+ T cells to produce an antitumor effect [68]. Regarding exosomes derived from diffuse large B cell lymphoma cells, DCs can stimulate clonal expansion of T cells by pulsing with these exosomes, increasing the secretion of IL-6 and TNF-α and reducing the production of immunosuppressive cytokines IL-4 and IL-10 [66].

### T lymphocyte cell-derived exosomes

Immunotherapy using genetically engineered T cells to express chimeric antigen receptor (CAR) is rapidly becoming a promising new therapy [69, 70]. T cells can be mainly divided into two types according to phenotype, with corresponding receptors on their surfaces and antigen specificity, including CD4+ helper T cells and CD8+ CLTs. Because of their unique functions and different surface antigens, CD4+ helper T cells can be further divided into several groups, including Th17 cells, regulatory T cells (Tregs), and follicular helper T cells (Tfhs), etc [71, 72]. CD8+ CTLs that bind directly to antigens via MHC I enhance cellular immunity against intracellular pathogens and malignant cells. Apart from the direct killing effects on tumor cells, activated CD8+ T cells can also eliminate tumor cells by releasing exosomes [73]. In an *in vivo* study with a mouse model of melanoma, intratumoral administration of activated CD8+ T cell-derived exosomes caused interruption of fibroblastic stroma-mediated tumor invasion and metastasis [74]. Although, most CTLs are low-affinity, high-affinity CTLs are considered more essential to the immune response due to their highly robust function and increased sensitivity to detection. A recent study has shown that in the presence of IL-12, high-affinity CTLs secrete exosomes that activate low-affinity CTLs that are important in the immunotherapy of cancer [75]. The exosomes from IL-12-stimulated CTLs also activate bystander CD8+ T cells to produce IFN-γ and granzyme B (GZB), ultimately destroying infected cells [76]. Other findings showed that CD63-expressed exosomes from T cells are known to carry specific miRNAs that regulate the immune response and immune system development, and play an important role in promoting intercellular APC–T cell communications [77]. CD63+ exosomes exert the same anti-infective properties as CD8+ T cells [78]. Thus, activated CD8+ T cell-derived exosomes can link cytotoxic T cells to targeted cells, and enhance CTL-based immunotherapy. However, FasL-expressed exosomes from activated CD8+ T cells unexpectedly promoted the metastasis of Fas-resistant tumor cells through the activation of ERK/NFκB signaling pathways [79]. Xie et al. further demonstrated the suppressive effect of T cell-derived exosomes on DC-mediated CTL responses and antitumor immunity through the downregulation of MHC I and FasL signaling [80]. In addition, exosomes from exhausted CD8+ T cells can be taken up by functional CD8+ T cells, thus impairing their activity and secretion of cytotoxic factors [81]. Thus, these paradoxical discoveries may allow us to better understand the detailed functions of CD8+ T cell-released exosomes under different circumstances and shed light on systematic studies of dysfunctional anticancer immunity.

CD4+ helper T cell surface markers are mainly CD4, which is activated or adjusted or assists in immune responses when combined with MHC II on the surface of APCs. The exosomes isolated from CD4+ helper T cells contain both exosome-associated proteins (LAMP-1, TCR and LFA-1) and CD4 T cell markers (CD4, TCR, LFA-1, CD25 and FasL) and participate in CTL responses and antitumor immunity [82]. Moreover, altered expression of bioactive messengers on CD4+ T cell-derived exosomes has been demonstrated to be the underlying pathogenic mechanism for some inflammatory diseases [83]. Along similar lines, these exosomes can interact with target cells via CD4-MHC interactions, and ultimately eliminate immunodeficient cells [84]. In addition, activated CD4+ helper T cell-released exosomes can also serve as a potent inducer for the activation of phagocytes and B cells, contributing to amplifying the inflammatory response [85, 86].

Recent studies have shown that Treg cells are responsible for negatively regulating the body's immune response and maintaining immunological tolerance [87], and CD4+CD25+ Treg cells are the most active cells in the current research. Recent findings suggest that Treg cells also control immune responses via the production of secreted exosomes. Treg-exosomes are reported to contain unique molecular cargoes of bioactive messengers (specific miRNAs and iNOS). Once delivered into target cells, these cargoes can block cell cycle progression, induce apoptosis [88, 89], and suppress CTL-mediated anti-cancer immunity [90]. A recent report conducted by Chen et al. showed that exosomes secreted by Treg cells, especially donor-type Tregs, are known to inhibit immunological rejection and create immune tolerance by impairing self-reactive CD8+ T cells during organ transplantation [91]. In particular, the expression of CD73 on Treg cell-derived exosomes is essential for their suppressive function [92]. These inhibitory effects on the immune system can be evidently reversed after treatment with GW4869, an exosome inhibitor [93].

### Natural killer cell-derived exosomes

As an important component of the innate immune system, NK cells contribute to immunosurveillance and function as the body's first-line of defense against several human disorders, including pathogen infections and cancers. NK cells can directly recognize and effectively kill oncogenic transformed cells that are normally devoid of class I MHC antigen expression, participating in anti-cancer immunity [94]. Recently, NK cells have also been proven to be involved in the control of the immune response using other methods independent of the cell activation status, one of which is via exosomes [95]. More importantly, exosomes derived from NK cells also harbor prototype NK markers (e.g., CD56) and killer proteins (e.g., FasL and perforin) [96]. Additionally, NK-exosomes can exert their cytolytic activity by directly diffusing into tumor tissues, and subsequently overcome the homing deficiency of NK cells to tumor sites [97]. Several studies were recently performed to investigate the profiles of NK cell-derived exosomes in cancer patients. In addition to exosome-specific markers (e.g., tsg101, CD81, CD63 and CD9), NK cell markers (e.g., NKG2D, CD94, perforin, granzymes and CD40L) were also expressed in NK-derived exosomes, which are both involved in cytotoxicity and immune response [98, 99]. These exosomes can induce target cell death by multiple killing mechanisms. Accordingly, after treatment with NK exosomes, both CHLA255 neuroblastoma cells and SupB15 leukemia cells showed significantly activated caspase-independent and caspase-dependent cell death pathways [100]. Furthermore, NK cell-derived exosomes strengthened the anti-cancer activity of CD56+ NK cells [98]. In addition, targeted delivery of tumor suppressors by NK-exosomes resulted in effective inhibition of tumorigenic potential and immune escape mechanisms [101]. The immunotherapeutic potential and tumor-targeting ability of NK-exosomes can be further improved after IL-15 priming of NK cells [102]. NK cells can be obtained from both autologous and allogeneic sources [103], providing more clinical applications for NK-exosomes. Taken together, these findings indicate that NK cell-derived exosomes can potentially be exploited in support of cancer immunotherapy. However, one question limiting their clinical applications remains to be answered: how can functional NK-exosomes be isolated on a large scale? To address this issue, Jong et al. recently conducted a polymer precipitation method to isolate a large quantity of NK-exosomes [104], which may lay the foundation for their future applications in the clinic.

### Myeloid-derived suppressor cell-derived exosomes

Myeloid-derived suppressor cells (MDSCs), a heterogeneous group of immature myeloid cells, have a remarkable capacity to suppress T/NK cell cytotoxicity and serve as a major obstacle in cancer immunotherapy [105, 106]. The therapeutic efficacy of inhibiting MDSCs by pharmacological agents in cancers has been well reviewed [107]. Recently, several reports have preliminarily described the immunosuppressive roles of MDSC-exosomes within the microenvironment in cancers and autoimmune diseases [108–110]. The cargoes present in MDSC-exosomes have been proven to be consistent with their involvement in MDSC-mediated immune suppression [111]. Notably, upon doxorubicin treatment, the improved MDSC-derived miR-126a+ exosomes could promote metastasis and therapeutic resistance in breast tumor-bearing mice [112]. Elimination of MDSC-exosomes fosters the anti-cancer immunotherapeutic response [113]. Nonetheless, additional detailed research should be conducted to evaluate the interaction between MDSC-exosomes and other tumor-infiltrating immune cells, and their relevance in cancer immunotherapy. A better understanding of the biological function of MDSC-released exosomes will be important for their future therapeutic applications in cancer patients.

### Tumor-associated macrophage-derived exosomes

In the tumor microenvironment, macrophages have the ability to suppress T cell function, thereby facilitating tumor immune escape [114]. However, tumor-associated macrophages (TAMs) often exert two opposing phenotypes: anti-tumorigenic M1 subtype and pro-tumorigenic M2 subtype [115]. Accumulating evidence indicates that TAMs also secrete exosomes to modulate multiple aspects of cancer biology and the immune response [116, 117]. Exosomes released from TAMs induce a Treg/Th17 imbalance by transferring miRNAs into CD4+ T cells, thus directly generating an immune-suppressive microenvironment and promoting ovarian cancer progression [118]. Recently, several studies have shown that TAM-exosomes with immunosuppressive activity are predominantly released from M2 subtype macrophages, and promote cancer progression and therapeutic resistance [119, 120]. Accordingly, M2-derived exosomes determine TAM-mediated promigratory activity by transferring functional apolipoprotein E into recipient gastric cancer cells [121]. M2 macrophage-derived exosomes also accelerate cancer cell migration, invasion and chemotherapy resistance by transferring oncogenic miRNAs [122, 123]. However, comprehensive molecular profiling and functional analysis have revealed that TAM-derived exosomes predominantly present Th1/M1 polarization signatures, and their cargoes enhance pro-inflammatory signaling and the immune response [124]. Furthermore, in a model of tumor-bearing mice, intravenous injection of M1 macrophage-derived exosomes can repolarize M2 to M1 macrophages in the microenvironment and significantly potentiate the anticancer efficacy of PD-L1 inhibitors [125]. In addition, these exosomes can act as transmitters to exchange components among other immune cells and to enhance the immune response. For example, Xu’s group demonstrated that these exosomes function as potential vehicles to convey phagocytosed antigens to DCs and finally strengthen T-cell responses [126]. Even though the immune-regulatory roles of TAM-exosomes require additional studies to clarify, these findings suggest that TAM-derived exosomes have the potential to increase anti-tumor immunity.

### Mast cell-derived exosomes

Mast cells (MCs) can secrete exosomes that display biological functions in RNA and protein transfer, intercellular communication and immunoregulation [127, 128]. It was pointed that MC-exosomes have been reported to destroy intestinal barrier function, which is attributed to exosome-carried miRNAs transferred to targeted cells [129]. Recent studies found that MC-derived exosomes can be taken up by lung cancer cells, and subsequently increase cancer cell proliferation by transferring KIT protein [130]. Morphological analysis about the effects of these exosomes on lung epithelial tumor cells revealed an epithelial to mesenchymal transition-like phenotype in exosome-recipient A549 cells. Transcript analysis further indicated that the EMT-associated phosphorylation cascades were obviously upregulated by MC-exosome treatment [131]. In addition, MC-derived exosomes can affect the biological functions of DCs, T cells and B cells. For example, CD63+ and OX40L+ exosomes from MCs promote the proliferation and differentiation of CD4+ Th2 cells via the OX40L-OX40 interaction [132]. MC-exosomes also induce immature DCs to upregulate MHC II, CD40, CD80, and CD86 molecules and to confer antigen-presenting capacity to T cells, thereby leading to the initiation of antigen-specific immune responses [133]. However, currently, the effect of MC-released exosomes on the anti-cancer immunity is still under investigation and might be a highly attractive topic in the future.

### Neutrophil-derived exosomes

Proteomic and RNA microarray analyses indicate that neutrophil-derived exosomes contain proteins, mRNA and miRNAs, which are associated with inflammatory reactions, immune response and cell communication [134–136]. Functional studies further discover that neutrophil-derived exosomes can affect the activity of other immune cells, such as macrophages, by transferring several proinflammatory factors [137]. These exosomes have been reported to bind and degrade ECM via integrin Mac-1 and neutrophil elastase (NE), consequently leading to inflammatory disease progression [138]. Conversely, Li et al. recently found that these exosomes significantly suppress the proliferation and migration of endothelial cells, thereby impairing pathological angiogenesis in immune disorders [139]. In addition, Vargas et al. preliminarily confirmed the tumor susceptibility gene 101 in neutrophil-derived exosomes [134]. However, to the best of our knowledge, no relevant studies have been conducted to explain the underlying molecular mechanisms of neutrophil-derived exosomes in the regulation of antitumor immune responses.

## Exosome-based immunotherapy in animal models

The potential of exosomes in the field of cancer immunotherapy is huge, and exosomes may become the most effective cancer vaccines as well as targeted antigen/drug carriers. Since exosomes can induce tumor-specific immunity, they have attracted wide attention as potential cancer vaccines, and animal and clinical trials have been conducted to verify their efficacy (Table 1). Recent studies have begun to expand our understanding of the role of TAEs in DC-mediated anti-cancer immune responses, and revealed the potential of TAEs as a new approach to cancer vaccines [140].

**Table 1:** The potential of exosomes as a new approach to cancer vaccines in animal models

Animal Models	Cancer	External stimulus	Exosome source	Clinical significance	Reference
BALB/c mice	None	Exposure to magnetic iron oxide nanoparticles	From alveolar region	Induce the maturation of DCs and activation of T cells	[142]
WEHI3B-bearing mice	Leukemia	Vaccination with TAE-loaded DC	TAE	Upregulate CD11c, MHC II and IL-12 in DC	[143]
Mouse plasmacytoma model	Plasmacytoma	Vaccination with a single dose (5 microg) of exosome protein	From plasmacytoma cells	Produce specific CTLs, induce tumor-specific immunity	[144]
C57BL/6 mice	Melanoma	Vaccination with CIITA-Exo	CIITA gene modified TAE	Trigger Th-1 type immune responses	[145]
BALB/c mice	Malignant mesothelioma	Vaccination with TAE-loaded DC	TAE	Increase median and overall survival of mice	[146]
Tumor-bearing mice	Melanoma and Lewis lung carcinoma	Vaccination with DEX bearing antigens from two types of tumor	DEX	Prevents both tumors growth in mice	[148]
B16-bearing mice	Melanoma	Vaccination with DEXs loaded with the iNKT-cell ligand αGC	DEX	Activate CD4+ and CD8+ T cells, increase the survival of mice	[149]

Note: *DC* dendritic cell. *TAE* Tumor-associated exosomes. *DEX* DC-derived exosomes. *CIITA* Class II transactivator. *αGC* α-galactosylceramide

TAEs can effectively act on DCs, thus inducing a stronger immune response and making up for the deficiency of DC immunotherapy [141]. After entering the systemic circulation, exosomes generated from BALB/c mice can transmit signals to the immune system, which can then induce the maturation of DCs and the activation of T cells [142]. In further research on tumor-bearing mice vaccinated with TAE-loaded DC, the TAEs were effectively ingested by DCs and subsequently upregulated the expression of CD11c, MHC II, and IL-12 [143].

Plasmacytoma cells release exosomes containing tumor antigens (P1A and intracisternal A particle protein) and HSP70 protein. They were used as a vaccine, and the vaccinated mice could produce specific CTLs, inducing tumor-specific immunity [144]. Exosomes derived from a CIITA (Class II transactivator) gene modified B16F1 murine melanoma cell line for use as a vaccine (CIITA-Exo) can express MHC II and tumor antigen TRP2. CIITA-Exo were injected into mice and they were confirmed to induce a Th1-polarization immune response, including upregulation of Th1 IgG2a antibodies, IFN-γ cytokines and TRP2 specific CD8+ T cells [145]. Exosomes derived from malignant mesothelioma cells can be used as an antigen source for DC-based immunotherapy, and tumor-bearing mice that received tumor exosome-loaded DC immunotherapy had higher survival rates [146]. However, since tumor-derived exosomes can not only stimulate the anti-tumor immune response but also promote immunosuppression and interfere with anti-tumor immunotherapy, it is necessary to understand the immune-stimulating mechanism of exosomes so that they can be used as adjuvants and antigenic components of anti-tumor vaccines [147].

In addition, effective dual exosome vaccines against melanoma (B16) and Lewis lung carcinoma (LLC) have also been developed to generate DEX carrying tumor antigens from B16 and LLC cells, which can inhibit the development of both tumors in mice after vaccination [148]. DEX loaded with the iNKT-cell ligand α-galactosylceramide (αGC) activates CD4+ T cells, OVA-specific CD8+ T and B lymphocytes, which then improves the survival rate and survival time in a B16 melanoma mouse model [149].

## Exosomes: effective markers for the adaptive immune activation of immunotherapy

Immunotherapy has become an important treatment choice for cancer patients. Currently, these existing biomarkers of immunotherapy are characterized by a low efficiency of responder stratification and high risk due to the need for invasive operations, so it is urgent to identify new biomarkers. For example, TAEs and CD3+ T cell-derived exosomes of head and neck squamous cell carcinoma patients who received a combination of cetuximab, ipilimumab, and radiotherapy, can replace immune cells to monitor the response of the patient to tumor therapy [150]. In addition for initiating immune responses and delivering drugs, exosomes have been found to be predictive markers for adaptive immune activation of immunotherapy [151, 152].

The activation of T and B cells in the adaptive immune response occurs in lymphoid tissues and is assessed primarily by evaluating the titer of serum antibodies and the responses of peripheral blood T lymphocytes. Exosomal PD-L1 is a potential early marker of adaptive immune activation after immunotherapy with PD-1 blocking antibodies in melanoma patients and predicts a clinical response [23]. Blocking the PD-1 pathway increased the production of IFN-γ by PD-1+CD8+ T cells, which in turn induced the expression of PD-L1 in various cells in the tumor microenvironment. In the early stages of immunotherapy in melanoma patients, there was a significantly higher increase in exosomal PD-L1 among responders, while there were no significant differences in other types of PD-L1, suggesting that exosomal PD-L1 is a marker of adaptive immune activation.

Studies have shown that activated lymphocytes release a large number of exosomes containing microRNAs, such as miR-150, and the microRNA characteristics of CD4+ T cell-derived exosomes are significantly different from intracellular microRNA characteristics in the same cells. After vaccination with adjuvant-OVA, the serum miR-150 level in normal mice increased significantly, to a level similar to that of immune mice that were depleted of mature CD4+ T lymphocytes. This suggests that when the immune system is activated after vaccination, the lymphocytes involved in the response will release a large number of easily detectable exosomes into the blood; thus, there are also easily measured levels of lymphocyte-derived exosomal microRNAs [153]. Similarly, plasma exosomal microRNAs from patients have been identified as potential biomarkers for immunotherapy of NSCLC. A controlled study of patients with advanced EGFR/ALK wild-type NSCLC who received PD-1/PD-L1 inhibitors showed that compared with normal controls, NSCLC patients had more than 150 differentially expressed exosomal microRNAs. Among them, it was found that low levels of exosome-derived hsa-miR-320d, hsa-miR-320c, and hsa-miR-320b may indicate the better efficacy of PD-1/PD-L1 immunotherapy in advanced NSCLCs. In addition, when hsa-miR-125b-5p, a T-cell suppressor in exosomes, is downregulated during immunotherapy, NSCLC patients may gain enhanced T-cell function and respond well [154].

## Exosomes: underlying targets for the regulation of cancer immunotherapy

The molecular mechanisms involved in targeting exosomes as cancer vaccines may provide important insights into immune recognition and therapeutic interventions [155]. More importantly, exosomes contain large amounts of tumor antigens such as MHC I and can be used as cell-free vaccines in cancer immunotherapy [156]. In the presence of APC, DC-derived exosomes have been reported to load multiple peptide antigens (e.g., MHC I, MHC II), and thereby stimulating both CD4+ helper T cells and CD8+ CLTs to participate in the anti-tumor response [157]. In a mouse model of pancreatic cancer, subcutaneous injection of TAE-DC vaccines significantly recovered the activated T cells in the tumor environment and improved the therapeutic effect [158]. Furthermore, vaccination within TAE-exosome loaded T cells (exosome-T) has the ability to counteract CD4+CD25+ Treg cell-mediated immunosuppression and to trigger long-term CTL memory, providing attractive strategies for inducing immune responses against human cancers [60, 159]. Similarly, the HER2-specific exosome-T vaccine was recently developed to efficaciously strengthen the patient’s immune system against HER2-positive breast cancer [160]. However, exosome-based strategies also have immunosuppressive effects and may alleviate the immune response against cancer by inducing apoptosis of activated CD8+ T cells to interfere with immunotherapy [161]. Even so, the use of exosome-vaccination for immunotherapy can still be considered by adjusting the delivery route, dose, and modification of targeted exosomes.

To improve the targeting of exosomes and overcome the limitations of autologous use, many studies have genetically engineered exosomes to express specific antigen molecules or target cancer cells to enhance anti-cancer immunogenicity [162]. For example, a new synthetic polyvalent antibody redirected exosome (SMART-EXO) was produced by using the transmembrane domain of human platelet-derived growth factor receptor to display two different types of monoclonal antibodies on the surface of an exosome. By targeting the CD3 receptor on the surface of T cells, SMART-EXOs with the breast cancer-related HER2 receptor and EGFR receptor can activate CTLs, which then exhibit highly potent and specific anti-tumor activity both *in vitro* and *in vivo* [163, 164]. In addition, antigens can also be artificially transfected into exosomes. For example, HEK293 cell-released exosomes can be transfected with EBV protein gp350 and thus activate T cells by expressing gp350 to target CD19 on B cells, providing a novel strategy for the immunotherapy of B lymphocytic leukemia [165]. To date, emerging studies have provided novel insights into the development of exosome-based drug delivery systems for cancer treatment. It should also be noted that because of their natural properties, exosomes are less toxic and immunogenic, and can serve as attractive carriers of cytotoxic agents, such as paclitaxel, docetaxel and doxorubicin, with better stability and higher specificity for targeted tumor cells [166–168]. Therapeutic agent-carried exosomes have the ability to exert dual inhibition of targeted tumor growth [169]. Currently, a dual-functional exosome-based superparamagnetic nanoparticle cluster (SMNC-EXO) has been developed using multiple superparamagnetic nanoparticles anchored to each exosome to form a cluster. Then in the presence of external magnetic fields, SMNC-EXOs have a powerful capability to deliver therapeutic drugs to targeted cancer cells [170]. Thus, it will be interesting to explore the possibility of exosome-associated technologies as potential therapeutic options for anti-cancer immunotherapy.

## Clinical implications

Based on extensive research into the role of exosomes in cancer immunotherapy and their relevance as diagnostic and therapeutic targets, a large number of clinical trials have been conducted with exosomes. Targeting TAE dysregulation pathways, such as the heparinase/syndecan-1 axis, is a new approach to cancer treatment in the context of the role of TAEs in promoting cancer cell survival and growth [171, 172]. Exosomes are also used as therapeutic markers in immunotherapy. In patients with malignant glioma receiving anti-survivin immunotherapy, the decreased release of CD9+/GFAP+/SVN+ and CD9+/SVN+ exosomes may be related to the prolonged progression-free survival of patients [173]. Furthermore, new evidence suggests that tumor cell-derived exosome DNA (ExoDNA) can also activate immune cells by STING/cGAS, and therefore, ExoDNA can both regulate tumor immunity and act as a key regulator of checkpoint immunotherapy [174].

The first exosome phase I trial conducted with vaccination of metastatic melanoma patients with autologous DEX verified the safety of exosome administration. However, since no specific CD4+ or CD8+ T cell responses were detected in the peripheral blood, it is still necessary to investigate the mechanism of vaccine antigen diffusion observed in this phase I trial [175]. In addition, the use of DEX in clinical trials of patients with NSCLC has been shown to mediate MAGE specific T cell responses and increase NK lysis activity [176]. DEX derived from blood cells in cancer patients has been shown to be safe and feasible for immunotherapy and has been successfully used in some small clinical trials, such as the phase II clinical trial in France of a DEX with a T-cell-dependent antitumor effect [177]. Even in the brain, which was previously thought to be able to block the entry of tumor-specific immune cells, DEX has been shown to be effective against glioma in mice, suggesting that DEX immunotherapy may be a new treatment for brain tumors [178]. DEX immunotherapy leads to a more precise and accurate immune response against tumor cells than other noncell-based therapies. Compared with other cell-based therapies, DEX immunotherapy has higher bioavailability and biostability, with higher yields and lower costs [179].

In ongoing clinical trials, exosomes are considered immunotherapeutic vaccines, markers of cancer diagnosis, prognosis, recurrence and metastasis, or drug delivery carriers for cancer treatment (Table 2). Exosomes as immunotherapeutic vaccines for cancer immunotherapy, including DEX combined with cyclophosphamide for NSCLC, TAEs combined with an antisense molecule against glioma, and mesenchymal stromal cell-derived exosomes with KrasG12D siRNA (iExosomes), were studied in pancreatic cancer. A large number of clinical trials have explored the possibility of using exosomes as diagnostic, prognostic and therapeutic markers for lung, prostate, renal cell, gastric, breast, gallbladder, pancreatic, and rectal cancers. The safety and efficacy of exosomes as curcumin carriers have been verified for the treatment of colorectal cancer in clinical trials. Therefore, based on the existing experimental data and clinical trials, exosomes are expected to become biomarkers, drug carriers and immunotherapeutic vaccines for a variety of cancers.

**Table 2:** The ongoing clinical trials of cancer immunotherapy based on exosomes

ID	Sponsor	Status	Cancer	Therapy strategy	Purpose
					**Immunotherapeutic vaccines**
NCT01159288	Gustave Roussy, Cancer Campus, Grand Paris	Completed	NSCLC	mCTX- and tumor antigen-loaded Dex	Dex are able to activate innate and adaptive immunity
NCT01550523	Sidney Kimmel Cancer Center at Thomas Jefferson University	Completed	Recurrent malignant gliomas	An antisense molecule designed to shut down a targeted surface receptor protein by TAEs	TAEs deliver tumor antigens, and activate the immune response
NCT03608631	M.D. Anderson Cancer Center	Not yet recruiting	Pancreas cancer	iExosomes	iExosomes may work better at treating Metastatic pancreatic cancer with KrasG12D mutation
					**Markers of cancer diagnosis and prognosis**
NCT03542253	Second Affiliated Hospital of Soochow University	Not yet recruiting	Early lung cancer	None	Exosomal microRNAs combined CT as early diagnostic markers
NCT03830619	Wuhan Union Hospital, China	Recruiting	Lung cancer	None	Exosomal lncRNAs as diagnostic markers
NCT03974204	Centre Oscar Lambret	Not yet recruiting	Breast cancer with leptomeningeal metastasis	None	Exosomes in the cerebrospinal fluid as diagnostic markers
NCT04182893	Shanghai Chest Hospital	Recruiting	Malignant pulmonary nodules	None	ctDNA and exosome RNA combined as diagnostic markers
NCT04053855	Centre Hospitalier Universitaire de Saint Etienne	Recruiting	Clear cell renal cell carcinoma	None	Urinary exosomes as early diagnostic markers
NCT03821909	The Affiliated Nanjing Drum Tower Hospital of Nanjing University Medical School	Recruiting	Pancreatic cancer	None	MicroRNA markers of exosomes from patients with primary tumors as diagnostic and prognostic markers
NCT01344109	Leo W. Jenkins Cancer Center	Withdrawn	Breast cancer	Neoadjuvant chemotherapy	TAEs as diagnostic and prognostic markers
NCT03911999	Chinese University of Hong Kong	Recruiting	Prostate cancer	None	Exosomal microRNAs as diagnostic and monitoring markers
NCT01779583	Hospital Miguel Servet	Unknown	Gastric cancer	None	TAEs as diagnostic, prognostic and predictive markers
NCT03102268	The Second Hospital of Nanjing Medical University	Recruiting	Cholangiocarcinoma	Surgery	Noncoding RNAs from TAEs as diagnostic, prognostic and predictive markers
NCT02439008	Centre Oscar Lambret	Terminated	Carcinoma	High-dose hypofractionated radiotherapy	Early markers of tumor response
NCT03874559	University of Kansas Medical Center	Recruiting	Rectal cancer	Neoadjuvant chemoradiation therapy	Pathologic response markers
NCT02862470	National Taiwan University Hospital	Active, not recruiting	Thyroid cancer	Lovastatin and Vildagliptin	Urine exosomes as prognostic markers and therapeutic targets
NCT03581435	Shanghai Jiao Tong University School of Medicine	Recruiting	Gallbladder carcinoma	None	Circulating exosome from blood specimens as prognostic and predictive markers
NCT02310451	Centre Hospitalier Universitaire de Nice	Unknown	Metastatic melanoma	Alkylating agents (temozolomide and fotemustine) or vemurafenib	Exosomes from senescent Melanoma cells as a prognostic factor and marker of therapeutic response
NCT03985696	University Hospital, Limoges	Recruiting	Non-Hodgkin B-cell lymphomas	Monoclonal anti-CD20 antibody, rituximab, in combination of CHOP chemotherapy	Immunotherapeutic targets (CD20, PD-L1) on exosomes from B-NHL contribute to therapeutic resistance
NCT02393703	Memorial Sloan Kettering Cancer Center	Active, not recruiting	Pancreatic cancer	None	Disease recurrence and outcomes markers
NCT03800121	Centre Georges Francois Leclerc	Recruiting	Sarcoma	None	Serum TAEs to monitor disease and predict recurrence
NCT03108677	Ruijin Hospital	Recruiting	Primary high-grade osteosarcoma with lung metastases	None	Circulating exosomal RNA as marker for lung metastases
					**Drug delivery carriers**
NCT01294072	University of Louisville	Active, not recruiting	Colon cancer	Curcumin	Plant exosomes as delivery vehicle for curcumin

Notes: The data source: https://clinicaltrials.gov/. *mCTX* Cyclophosphamide. *iExosomes* Mesenchymal stromal cells-derived exosomes with KrasG12D siRNA

## Conclusions

Although exosomes are a relatively new area of research, there has been widespread interest in the field of cancer therapy regarding the potential use of exosomes as new low-toxicity inhibitors in immunotherapy, as potential cancer markers, or as a safer and more efficient method of delivering anti-cancer drugs. Exosomes, a kind of small extracellular vesicle, can be released by tumor cells or immune cells into the extracellular environment. Increasing studies have led to more recent updates to the evidence suggesting that exosomes can display immunomodulatory properties and operate as potential therapeutic agents. Moreover, exosomes exhibit important functional roles in transferring proteins, nucleic acids, and lipid contents, consequently contributing to intercellular communication and immune regulation [132, 180]. More importantly, some of these biologically active cargoes on exosomes, such as MHC and costimulatory molecules, have been proven to participate in exosome-mediated anti-cancer immune responses. To date, cumulative studies have demonstrated that the exosome-mediated immune response is dependent on the functional link between several immune cells and tumor cells. Thus, a better understanding of the cell-specific molecular events on exosomes would be helpful to pave the way for developing novel potential exosome-based biomarkers and therapeutics. Recent advances in clarifying the molecular and functional profiles of exosomes have also led to the development of increasingly effective agents that might be potentially used in cancer immunotherapies.

Even though exosome-based strategies have been demonstrated to enhance the anti-cancer immunotherapy, the evidence regarding their clinical application in cancer patients has yielded only modest benefits. In particular, there are still some difficulties in the separation, production, biocompatibility and manufacturing practices of exosomes before clinical realization of their full potential [181, 182]. First, most exosomes are currently isolated from cell culture supernatants and complex biological fluids (such as plasma); thus, the production and purity of exosomes are limited [183]. When using exosomes as immunotherapy or for other approaches, large-scale stable preparation methods must be achieved. Although some studies have reported protocols for mass production of exosomes and improvements in biocompatibility [184, 185], further preclinical and clinical studies are needed for validation. Furthermore, exosome-based immunotherapy is still in the early clinical trial stage at present, and there are no specific international guidelines for the management of the production and application of this new type of therapeutic agent [17, 186]. Therefore, before exosomes are officially used in the clinic, the quality classifications and standards for biopharmaceuticals should be addressed, and there is a need to develop specific GMP guidelines as soon as possible to ensure the safety of exosomal treatment.

## Data Availability

Not applicable.

## Abbreviations

PD-1: Programmed cell death 1
PD-L1: Programmed cell death 1 ligand 1
TGF-β: Transforming growth factor-β
DC: Dendritic cell
MHC I: Major histocompatibility complex class I molecule
NK: Natural killer
TAE: Tumor-associated exosome
CTL: Cytotoxic T cell
CIK: Cytokine-induced killer cell
MHC II: Major histocompatibility complex class I molecules
IL: Interleukin
TNF: Tumor necrosis factor
IFN: Interferon
APC: Antigen presenting cell
DEX: DC-derived exosome
AFP: α-Fetoprotein
CAR: Chimeric antigen receptor
Tregs: Regulatory T cells
Tfhs: Follicular helper T cells
MDSCs: Myeloid-derived suppressor cells
MCs: Mast cells
LLC: Lewis lung carcinoma
αGC: α-Galactosylceramide
SMART-EXO: Synthetic polyvalent antibody redirected exosome
SMNC-EXO: Exosome-based superparamagnetic nanoparticle cluster
